# Unlocking the Therapeutic Potential of Patchouli Leaves: A Comprehensive Review of Phytochemical and Pharmacological Insights

**DOI:** 10.3390/plants14071034

**Published:** 2025-03-26

**Authors:** Isack Ibrahim Mrisho, Elshan Musazade, Haobo Chen, Huixuan Zhao, Junjia Xing, Xue Li, Jiahong Han, Enbo Cai

**Affiliations:** 1 College of Chinese Medicinal Material, Jilin Agricultural University, Changchun 130118, China; issackmson98@gmail.com (I.I.M.); 18293288363@163.com (H.C.); 13356295853@163.com (H.Z.); hill_0307@163.com (J.X.); lixue_20005221@126.com (X.L.); 2 Key Laboratory of Soybean Molecular Design Breeding, Northeast Institute of Geography and Agroecology, Chinese Academy of Sciences, Changchun 130102, China; elshan.musazade1@gmail.com; 3 College of Life Science, Jilin Agricultural University, Changchun 130118, China

**Keywords:** medicinal plant, patchouli (*Pogostemon cablin*), patchouli leaves, phytochemistry, pharmacological activities

## Abstract

Plant-based products play an increasingly vital role in the pharmaceutical industry, including *Pogostemon cablin* (Blanco) Benth. (patchouli), which is notable for its rich history and extensive use in traditional medicine. Patchouli has a longstanding historical use as a remedy for a wide range of health conditions, including colds, fevers, headaches, inflammation, digestive disorders, and insect and snake bites. Comprehensive phytochemical studies have revealed that patchouli leaves contain diverse valuable bioactive compounds, notably patchouli alcohol, β-patchoulene, pogostone, α-bulnesene, and β-caryophyllene. Recent studies have demonstrated that patchouli leaves exhibit various pharmacological properties, including anti-oxidant, anti-inflammatory, antimicrobial, antidepressant, and anticancer effects. Despite robust traditional knowledge, specific therapeutic applications of patchouli leaves require scientific validation and standardization of their bioactive compounds. This review provides a comprehensive overview of the existing literature on the phytochemical composition, pharmacological properties, and underlying mechanisms of action of patchouli essential oil (PEO) and plant extracts obtained from patchouli leaves. It offers detailed insights into potential therapeutic applications, aiming to inform and guide future research across multiple medical disciplines. Ultimately, this review underscores the need for further research to validate and develop the medicinal applications of patchouli leaves, providing a foundation for future healthcare advancements.

## 1. Introduction

Medicinal and aromatic plants represent a significant component of the world’s flora and serve as essential sources of raw materials for industries, such as fragrances, cosmetics, food flavoring, and pharmaceuticals [1,2]. These plants have been used in herbal medicine for centuries to address various human health issues. Despite advancements in synthetic pharmaceuticals, the global demand for herbal medicines continues to increase, underscoring their enduring significance and therapeutic potential [1,2,3]. Notably, many contemporary pharmaceutical drugs are derived from plant-based compounds, with traditional plant uses enhanced through modern technological applications [4].

Among the various medicinal species, *Pogostemon cablin* (Blanco) Benth. (patchouli), a member of the Lamiaceae family, stands out for its extensive use in traditional medicine across South and Southeast Asia [5,6]. Its therapeutic applications are well documented in Indian and Chinese medicine, where it addresses conditions such as exterior syndrome, relieving dampness, counteracting summer heat, and acting as an appetite stimulant and antiemetic [7,8,9]. In traditional Chinese medicine, patchouli is integral to formulations such as Pogostemon Herba and various pills, which are used for their anti-inflammatory properties [10,11]. The medicinal applications of patchouli are widespread in countries such as China, Japan, and Malaysia, where it is used to treat ailments such as diarrhea, colds, headaches, and snake and insect bites [12,13,14].

Patchouli leaf is distinguished by its rich profile of bioactive compounds, including terpenoids (monoterpenoids, sesquiterpenoids, and triterpenoids), flavonoids, lignins, phytosterols, organic acids, glycosides, aldehydes, alcohols, and pyrone [15,16,17]. Among these, the essential oil extracted from its leaves, commonly known as patchouli essential oil (PEO), represents the most prominent and extensively studied component, highly valued for its distinctive spicy aroma and extensively utilized in perfumery and aromatherapy [7,18,19]. Beyond its aromatic qualities, PEO exhibits a broad spectrum of pharmacological activities, including sedative, anti-inflammatory [20], anti-oxidant, astringent, anti-mutagenic, antidepressant, diuretic, and antiseptic [21,22]. These activities are primarily attributed to key constituents, such as patchouli alcohol (patchoulol), pogostone, α-, and β-patchoulene, α-, and δ-guaiene, β-caryophyllene, *trans*-caryophyllene, and β-elemene [23,24,25]. Additionally, the complex composition of PEO contributes to its diverse therapeutic potential, including antibacterial [26,27,28], anti-insecticidal, anti-allergic [29], immunomodulatory, antithrombotic [30], antiviral, anticancer, cytotoxic, and anti-mutagenic effects [22,31].

The unique properties of the patchouli plant make it a valuable commercial crop with diverse industrial applications globally [9,18,32]. This review analyzes the phytochemical composition, pharmacological activities, and mechanisms of action of patchouli plant extracts derived from patchouli leaves. The goal is to guide future research across various medical disciplines by offering detailed insights into potential therapeutic applications. This review also underscores the need for further studies to validate and expand the medicinal uses of patchouli leaves and establish a foundation for future healthcare innovations. Overall, it highlights the potential for developing innovative, effective, and sustainable therapeutic agents by bridging traditional medicine with modern pharmacology. To gather relevant information on *P. cablin* (Blanco) Benth. leaves, we conducted a literature search spanning the last 20 years using PubMed, Scopus, and Web of Science (WoS) with the keywords “*Pogostemon cablin*”, “Patchouli”, “phytochemistry”, and “pharmacological activity”. Additional articles were retrieved through Google Scholar and by screening references. Studies not directly related to *P. cablin* leaves or lacking sufficient methodological detail were excluded.

## 2. Botanical Description

### 2.1. Taxonomy

- Kingdom: Plantae
- Family: Lamiaceae
- Genus: Pogostemon
- Species: *Pogostemon cablin* (Patchouli)

*P. cablin* (Blanco) Benth., commonly referred to as patchouli, is a member of the Lamiaceae family, which is one of the most prominent families of flowering plants, comprising 12 subfamilies, 240 genera, and approximately 7200 recognized species [33,34]. The Pogostemon genus, which includes approximately 80 species, is primarily native to Southeast Asia, with 20 species also found in India [35].

### 2.2. General Habitat and Distribution

Patchouli is a tropical plant well suited to warm climates, originally native to the Philippines but now widely cultivated in several Asian countries, including China, Indonesia, Vietnam, India, and Malaysia, as well as in regions such as Brazil, Singapore, Seychelles, and West Africa [6,9,36]. It thrives under specific conditions: temperatures between 24 °C and 28 °C, relative humidity of approximately 75%, annual rainfall of 2000 to 3000 mm, and altitudes ranging from sea level to 1200 m [37].

### 2.3. Morphology

*P. cablin* is a dicotyledonous plant species [38]. Morphologically, it is a perennial, and bushy herb that is well-suited to hot and humid climates. The species typically attains a height of 1–1.2 m, exhibiting an upright stem and ovate stalked leaves measuring approximately 10 cm in length and 2 cm in width [39]. The leaf margins are slightly lobed, and lobs have crenate–serrate teeth [9]. The lobes and apex of the leaves are obtuse. On the undersurface of the leaves and along the ribs, numerous trichomes on the epidermis serve as the primary accumulation sites for PEO [40]. The roots of patchouli are extensive and branched. The roots can penetrate a considerable depth in mature plants that can grow without interference. The plant produces small flowers that are pale pinkish-white in color [41,42]. Leaves, flowers, and seeds freely leave their aromas [35].

## 3. Phytochemical Composition of Patchouli Leaves

In recent years, research and development have focused on the *P. cablin* plant as a whole [43,44]; however, studies specifically addressing *P. cablin* leaves remain relatively scarce. The leaves contain a variety of phytochemical constituents, including volatile and non-volatile compounds [45]. These include monoterpenes, sesquiterpenes, flavonoids, organic acids, glycosides, phenolic compounds, and other chemical constituents [42,45].

### 3.1. Volatile Chemical Composition

**Figure 1:**
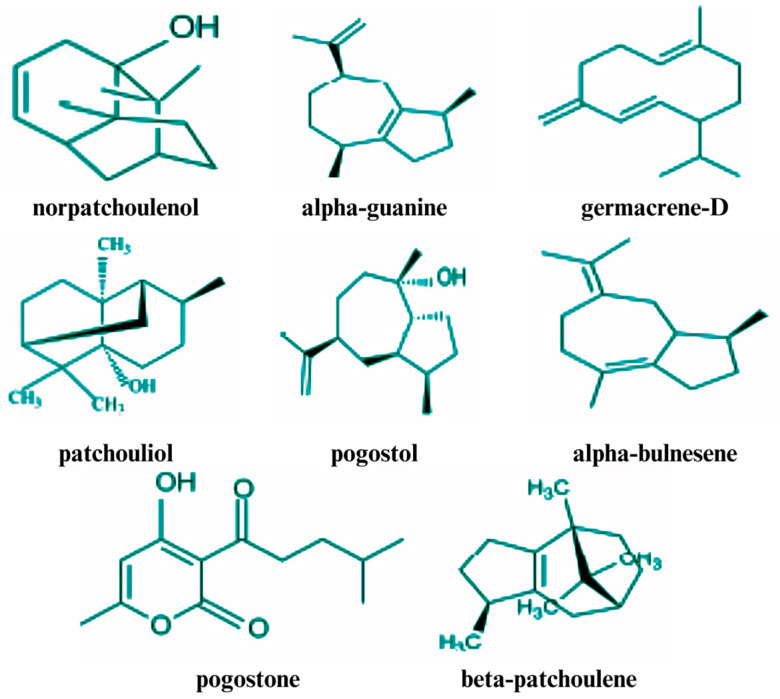
Structures of some of the volatile chemical compounds in patchouli leaves.

One of the main compounds extracted from patchouli leaves is PEO, which is well-known for its extensive range of pharmacological properties [46]. Several studies have investigated the various compounds present in this PEO [47], resulting in the identification of over 150 different compounds [48]. The chemical composition of PEO, including the volatile compounds, is detailed in Table 1, and the structures of several key volatile compounds are depicted in Figure 1.

PEO is composed of different phytochemicals, such as sesquiterpenes, ketones, alcohols, and other compounds [60]. However, sesquiterpenes are considered to be the main constituents of PEO [46]. Sesquiterpenes found in PEO include patchoulol (a tricyclic sesquiterpene), pogostone, pogostol, α-, β-, and δ-guaiene, α- and β-bulnesene, α-, β-, γ-, and δ-patchoulene, *cis*/*trans*-caryophyllene, and norpatchoulenol. Among these, patchoulol and patchoulene are present in the highest concentrations [61,62,63]. The biological activities of PEO are primarily determined by its constituent compounds, including pogostone, patchoulol, and α- and β-patchoulene [12,43]. Many researchers have identified patchoulol and α-patchoulene as key constituents that significantly influence the quality of PEO [12,64]. Additionally, the distinctive fragrance of PEO is primarily attributed to compounds such as pogostone and patchoulol, along with other sesquiterpene hydrocarbons, such as patchoulene, guaiene, and seychellene, which also play significant roles in defining its aromatic profile [43].

The chemical composition of PEO is highly variable and influenced by factors such as geographic location, environmental conditions, harvest timing, and processing techniques [46,65]. A study focusing on PEO from different regions in China identified nine primary components: patchoulol, pogostone, caryophyllene, α-, β-, and δ-guaiene, β-patchoulene, seychellene, and spathulenol [52]. Furthermore, analysis of leaves harvested from Hainan, China, between June and August revealed PEO contents of 0.8%, 0.7%, and 0.6%, respectively, with the highest concentration of patchoulol observed in June. Furthermore, gas chromatography-mass spectrometry (GC/MS) analysis of PEOs collected over a 24-h period revealed the presence of various compounds, including 1-octen-3-ol, α-guaiene, and β-patchoulene. However, it showed no significant compositional changes associated with harvest timing [59].

GC and GC/MS analyses revealed distinct profiles of PEO from different regions. In China, PEO from Gaoyao County in Guangdong Province was characterized by patchoulol (37.53%), patchouli ketone (pogostone) (21.31%), *trans*-caryophyllene (6.75%), α-guaiene (6.18%), and seychellene (1.99%) [66]. In Leizhou County, the PEO contain a variety of sesquiterpenes, including patchoulol, α-patchoulene, α-guaiene, aciphyllene, seychellene, and *trans*-caryophyllene [67]. Additional studies from Zhanxiang reported patchoulol (48.77%), α-guaiene (15.31%), δ-guaiacol (5.17%), α-guaiacol (4.52%), and patchoulone (0.18%), while research in Shipai noted patchoulol (60.59%), δ-guaiacol (6.14%), α-guaiacol (5.53%), and patchoulone (0.14%) [68].

In Vietnam, GC and nuclear magnetic resonance (NMR) analyses of patchouli leaves identified patchoulol as the major component at 32%, followed by α-guaiene at 14.9%, α-bulnesene at 14.6%, seychellene at 8.2%, and α-patchoulene at 5.5%, with another form of α-patchoulene at 5.2% [14,49]. Patchoulol was identified as a more odor-intensive component comprising 32–37% of the PEO [14,46]. In contrast, research in the Philippines indicates that the distinctive aroma of PEO is primarily attributed to germacrene-B, a major sesquiterpene component [69]. Additional volatile compounds identified in PEO include pogostol, δ-cardinene, daucosterol, germacrene-B, and three sesquiterpene hydroperoxides: 1α-hydroperoxyguaia-10(15),11-diene, 10α-hydroperoxyguaia-1,11-diene, and 15α-hydroperoxyguaia-1(10) [41].

In India, GC analysis identified patchoulol as the major component at 57.7%, followed by α-guaiene (13.2%), α-bulnesene (10.4%), seychellene (3.8%), (E)-caryophyllene (3.2%) α-humulene, (1.9%) norpatchoulenol, (1.8%) β-patchoulene, (1.7%), aciphyllene (1.6%), and γ-patchoulene (1.6%) [70]. In Indonesia, GC and GC/MS analyses of PEO have revealed a diverse array of compounds, including monoterpenes, such as α-, β-pinenes, and limonene. Among the sesquiterpenes identified were β-caryophyllene, α-humulene, α-copaene, δ-patchoulene, γ-gurjunene, germacrene D, α-, and δ-guaiene, β-, δ-elemene, seychellene, 7-epi-α-selinene, aciphyllene, and cycloseychellene. Additionally, several patchoulene isomers were identified, including α-, β-, γ-, and δ-patchoulene. Other terpenoids and related compounds identified include patchoulol, pogostol, isopatchoulenone, 9-oxopatchoulol, patchoulenone, caryophyllene oxide, norpatchoulenol, and nortetrapatchoulol [71]. In Brazil, GC/MS analysis identified 29 volatile compounds, including patchoulol (33.25%), pogostol (6.33%), seychellene (6.12%), norpatchoulenol (5.72%), α-bulnesene (4.11%), α-guaiene (2.99%), β-elemene (0.88%), and β-patchoulene (0.46%) [72].

The comparative analysis indicates that, while PEOs from China, Vietnam, India, Indonesia, and Brazil share several common compounds, such as patchoulol, α-guaiene, and seychellene, notable differences exist in their compositions and concentrations. Chinese PEOs exhibit significant regional variation, with some areas, such as Shipai County, producing oils with high levels of patchoulol (up to 60.59%) and unique compounds, like patchouli ketone. Vietnamese PEO is characterized by a balanced composition, with substantial amounts of patchoulol (32%), α-guaiene (14.9%), and α-bulnesene (14.6%). Indian PEO stands out for its exceptionally high patchoulol content (57.7%). Indonesian PEO contains a diverse range of compounds, including α-pinene, limonene, and δ-guaiene, which contribute to its distinct profile. Brazilian PEO, in contrast, features significant amounts of patchoulol (33.25%), pogostol (6.33%), and α-bulnesene (4.11%), along with a unique set of volatile compounds. These variations underscore the influence of geographical and environmental factors on the chemical composition of PEOs across different regions.

### 3.2. Non-Volatile Chemical Composition

**Figure 2:**
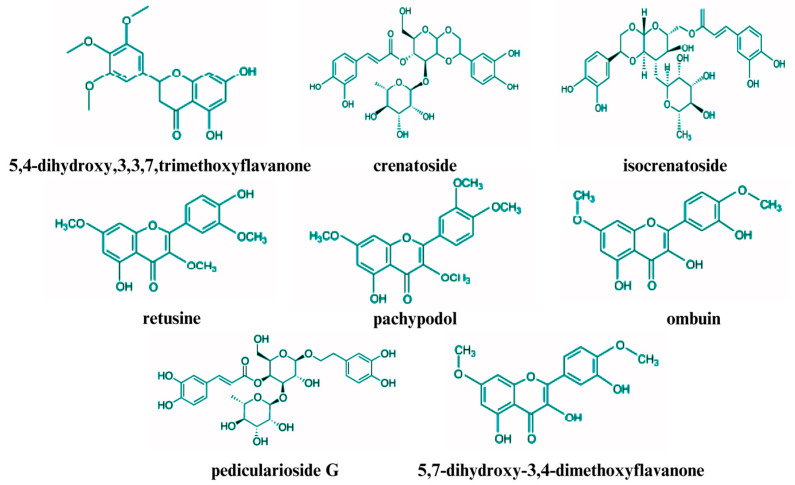
Structures of some non-volatile chemical compounds in patchouli leaves.

Patchouli leaves are abundant in non-volatile compounds, with over 50 such compounds identified and characterized through various analytical methods [14]. These compounds encompass a range of chemical classes, including flavonoids, lignans, non-volatile terpenoids (e.g., triterpenoids), glycosides, organic acids, and higher molecular weight aldehydes [48,70]. The non-volatile compounds of PEO are detailed in Table 2, and the structures of several key non-volatile compounds are depicted in Figure 2.

Studies have identified a variety of flavones and other non-volatile compounds in the aerial parts of the patchouli plant, particularly in the leaves [79]. These compounds include ombuin, licochalcone A, 3,3′,4′,5,7-pentahydroxyflavone, 4′,5,7-trihydroxyflavone (a derivative of apigenin), 5,7-dihydroxy-3′,4′-dimethoxyflavone, 5-hydroxy-3′,4′,7-trimethoxyflavone, 5-hydroxy-3′,4′,7-trimethoxyflavanone, 5-hydroxy-4′,7-dimethoxyflavone, 3,5-dihydroxy-4′,7-dimethoxyflavone, and 4′,5-dihydroxy-7-methoxyflavone [22].

In addition to these flavones, numerous studies have identified other significant non-volatile compounds in patchouli leaves, including pachypodol, retusine, stigmasterol, tilianin, dibutyl phthalate, and tschimganical A [80]. Pachypodol has garnered attention owing to its diverse biological activities [81]. Furthermore, patchouli leaves are rich in various glycosides, including phenylethanoid glycosides, such as verbascoside and pedicularioside G, and other glycosides, such as actinosides, campeoside, and isocrenatoside [14]. Recent research has also identified two novel glycosidic epimers, cablinosides A and B [82].

Methanol, ethanol, and hexane extracts of patchouli leaves have been extensively studied and have revealed a wide array of non-volatile chemical compounds. Through column chromatography and spectral analysis, researchers have successfully isolated nine distinct compounds: apigenin-7-O-β-D-(-6″-p-coumaroyl)-glucoside, 3″-O-methylcrenatoside, isocrenatoside, crenatoside, epifriedelinol, 5-hydroxymethyl-2-furfural, succinic acid, β-sitosterol, and daucosterol [83]. Additionally, high-speed countercurrent chromatography (HSCCC) and preparative high-performance liquid chromatography (HPLC) facilitated the isolation of five more compounds, four of which were identified as flavonoids: 5-hydroxy-7,3′,4′-trimethoxyflavanone, 5-hydroxy-3,7,3′,4′-tetramethoxyflavanone, 4′,5-dihydroxy-3′,7-dimethoxyflavanone, and 5,4′-dihydroxy-3,7,3′-trimethoxyflavanone [84]. Advanced analytical methods, such as HPLC-Q-TOF-MS, HPLC, and Thin-layer chromatography (TLC), have been employed to explore non-volatile components further, leading to the identification of compounds such as 7R-campeoside II, 7S-campeoside II, osmanthuside B, 2″,3″-O-acetylmartynoside, and 3′-methoxyisocrenatoside [62,75,76]. Zhou et al. (2013) [85] isolated 13 compounds from the aerial parts of patchouli using column chromatography and spectroscopic techniques. Notable compounds include methyl oleanolate, (−)-guaiacylglycerol, stigmast-4-ene-3-one, oleanolic acid, and 5α-stigmast-3,6-dione [85]. Furthermore, an HPLC-Diode Array Detection (HPLC-DAD) method was developed for the precise quantification of non-volatile components, including eight distinct flavonoids: ombuine, rhamnetin, 5-hydroxy-7,3′,4′-trimethoxyflavanone, 5-hydroxy-3,4′,7-trimethoxyflavone, 3,5-dihydroxy-7,4′-dimethoxyflavone, 4′,5-dihydroxy-3,3′,7-trimethoxyflavone, and 5-hydroxy-3,3′,4′,7-tetramethoxyflavone [74]. These studies collectively highlight the diverse chemical compositions of patchouli leaves and the advanced analytical techniques used to identify and quantify these compounds.

## 4. Pharmacological Activities

**Figure 3:**
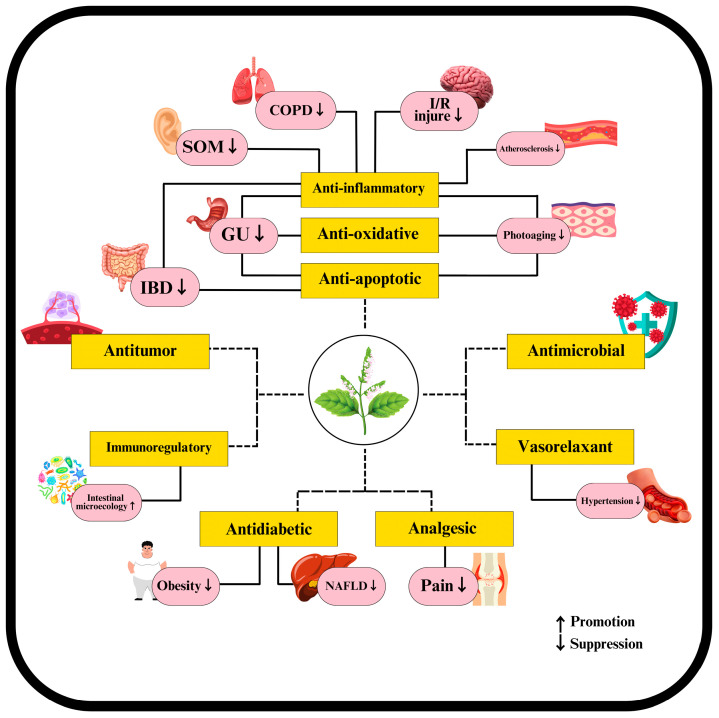
The pharmacological activities of chemical constituents in patchouli leaves.

Patchouli leaves display various pharmacological activities attributed to their diverse bioactive compounds. Extensive experimental studies have investigated the pharmacological effects of these compounds, broadly summarized in Figure 3 and detailed separately as volatile and non-volatile in Table 3 and Table 4. These activities include antimicrobial properties, where patchouli leaves demonstrate significant antibacterial, antifungal, and antiviral effects, particularly against influenza virus and HIV/AIDS [39,86,87]. In anticancer and tumor-related activities, patchouli leaf compounds inhibit cancer cell proliferation, induce apoptosis, and modulate the immune system, enhancing its ability to combat tumors and cancer cells [88,89]. Gastrointestinal protective activities are also notable, with patchouli leaves providing protection against gastrointestinal issues, antiemetic effects, regulation of defecation and constipation, anti-peptic ulcer properties, and anti-diarrheal benefits [90,91]. Additionally, patchouli leaves influence blood-related activities by promoting blood coagulation and fibrinolysis and exerting antithrombotic and antihypertensive effects [43,92]. The general protective and therapeutic activities of patchouli leaves include anti-oxidant, analgesic, anti-inflammatory, anti-mutagenic, and insecticidal properties, as well as benefits in treating skin diseases, aromatherapy, and aphrodisiac applications [93,94]. The metabolic and obesity-related activities of patchouli leaves include effects on obesity, intestinal microecology, and diabetes, further highlighting their comprehensive therapeutic potential [95,96].

### 4.1. Antimicrobial Activities

#### 4.1.1. Antibacterial Activities

Studies have demonstrated the antimicrobial properties of patchouli leaf extracts, effective against both Gram-negative and Gram-positive bacteria [39,86]. Punithavathy (2012) and Chuwen et al. (2013) showed that ethyl acetate (PCLE) and hexane (PCLH) extracts inhibit the growth of *Klebsiella pneumoniae* and *Staphylococcus aureus* in a concentration-dependent manner, with PCLE being more potent, particularly effective against *K. pneumoniae* at 0.03 mg/mL [127,128]. TLC of ethanol extracts identified terpenes, phenolic acids, and flavonoids, which inhibited the growth of *Escherichia coli*, *Erwinia* sp., *S. aureus*, and *Xanthomonas campestris* [129]. Aqueous patchouli extracts have also demonstrated enhanced resistance against *Moraxella catarrhalis*, *S. aureus*, and *Vibrio harveyi*, inhibiting biofilm formation by multidrug-resistant *V. harveyi* at a minimum inhibitory concentration (MIC) of 31.25 mg/mL through modulation of biofilm-associated genes [80,130].

PEO exhibits broad-spectrum antibacterial activity, including against *Acinetobacter baumannii*, *Aeromonas veronii*, *Candida albicans*, *Enterobacter aerogenes*, *Enterococcus faecalis*, *E. coli*, *K. pneumoniae*, *Pseudomonas aeruginosa*, *Salmonella enterica*, and *S. aureus* [28,103]. It is particularly effective against methicillin-resistant *S. aureus* (MRSA), with patchoulol and hexane extracts from dried patchouli leaves (Pogostemonis Herba) showing superior antibacterial efficacy [13,46]. PEO also demonstrates vigorous bactericidal activity against *Campylobacter jejuni*, *Listeria monocytogenes*, and *Shigella flexneri* [131]. One of the main constituents, pogostone, demonstrated antibacterial activity with a MIC of 4 µg/mL against *S. aureus*, disrupting bacterial cell membranes by interacting with membrane proteins [80,132]. Additionally, PEO resolves middle ear infections caused by *M. catarrhalis*, reducing inflammatory cell infiltration with MICs of 0.21 mg/mL and 0.026 mg/mL [80].

Patchoulol, a key compound in PEO, has potent antimicrobial effects, especially against *Helicobacter pylori*, which is linked to gastritis, peptic ulcers, and gastric cancer [104,133]. It targets *H. pylori* while preserving normal gut microbiota, reducing the risk of bacterial resistance [134]. Patchoulol inhibits *H. pylori* adhesion, motility, and urease activity by downregulating acid resistance genes, thus decreasing urease production [135]. It also protects gastric epithelial cells by preserving mitochondrial function, reducing oxidative stress, and activating anti-oxidant enzymes. Patchoulol’s anti-inflammatory effects modulate NLRP3 and NF-κB inflammasome pathways, and reduce neutrophil recruitment, cytokine production, and *H. pylori* survival within intracellular lysosomes [42,136].

#### 4.1.2. Antifungal Activities

PEO has been evaluated for its antifungal properties against the pathogenic fungi *Aspergillus niger* and *C. albicans*, both in vitro and in vivo, showing significant antifungal activity [28,86,103]. Patchoulol, a major constituent of PEO, has also demonstrated strong efficacy against [103]. Additionally, pogostone, another compound in PEO, and its synthetic analogs exhibit potent antifungal activity against *C. albicans* [131,137]. Li et al. provided compelling evidence for the therapeutic potential of pogostone, particularly in treating vulvovaginal candidiasis caused by *Candida* infections [138].

Furthermore, TLC analysis of the ethanol extract from the patchouli plant using solvents, such as ethanol, chloroform, and ether, identified the presence of terpenes, phenolic acids, and flavonoids. This extract exhibited significant antifungal activity, effectively inhibiting the growth of *C. albicans* and *Fusarium oxysporum* [129].

#### 4.1.3. Antiviral Activities

##### Anti-IFV Treatment of HIV/AIDS and Opportunistic Infections

Herbal medicines, such as patchouli, have been studied for their antiviral properties, particularly against influenza. Methanol extracts of patchouli leaves have shown potent antiviral activity, inhibiting the influenza A/PR/8/34 (H1N1) virus by up to 99.8% at 10 μg/mL with an IC50 of 2.6 μM [139]. Patchoulol, a compound in patchouli, also demonstrates significant anti-influenza effects in vivo. In mouse models, patchoulol administration (20–80 mg/kg for 7 days) improved survival rates, reduced viral loads, and modulated immune responses, including T cell levels, antibodies, and cytokine concentrations [35]. Patchoulol increased serum titers of anti-influenza IgA, IgM, and IgG antibodies, as well as CD3+, CD4+, and CD8+ T cells, while also reducing lung inflammation through the regulation of cytokines, such as TNF-α, IL-10, and IFN-γ [140,141].

Wu et al. further confirmed the antiviral efficacy of patchoulol, demonstrating its activity against influenza A (H2N2) with an IC50 of 4.0 μM, supporting its potential as an anti-influenza agent [140]. Additionally, a study found that patchoulol enhanced the innate immune response while suppressing the inflammatory factor IFN-α, reducing inflammation [14]. PEO has also been used in managing HIV/AIDS, particularly for treating opportunistic infections [142,143].

### 4.2. Anticancer and Tumor-Related Activities

#### 4.2.1. Anticancer Activity

Studies on patchouli have identified several bioactive compounds, including betulinic acid, moronic acid, and pachypodol, which show notable anticancer properties across various cancers, such as oral, lung, breast, colorectal, and pancreatic cancers. These compounds primarily exert their effects through antiproliferative and pro-apoptotic mechanisms [87,89].

Pachypodol, a key bioflavonoid from patchouli, has shown mild cytotoxicity in colon cancer cells, with an LD50 of 185.6 µM for cytotoxicity and 435.8 µM for general toxicity, indicating its potential to inhibit colon cancer cell growth [144]. Other phytochemicals from patchouli also demonstrate antitumor properties, further supporting its therapeutic potential in cancer treatment [145,146].

Patchoulol, another major compound in patchouli, exhibits significant anticancer activity, particularly against colorectal cancer. It inhibits cell proliferation, induces G1 phase arrest in CaCo-2 cells, and reduces tumor growth in animal models following dextran sodium sulfate (DSS)-induced tumorigenesis [147]. Patchoulol targets histone deacetylase 2 (HDAC2) and c-Myc oncogenes and activates NF-kB transcription factors to promote apoptosis, further suppressing tumor growth [148]. It also modulates key cell cycle regulators, including p21, cyclin D1, and CDK4, contributing to its anticancer effects.

Patchoulol’s anticancer effects extend to multiple mechanisms, including autophagy induction and modulation of the Akt/mTOR and EGFR-MAPK pathways, which are crucial for cancer cell proliferation [149]. In non-small cell lung cancer (NSCLC) models, patchoulol reduces cell viability by inducing cell cycle arrest and promoting oxidative DNA damage, as evidenced by increased levels of reactive oxygen species (ROS) and the DNA damage marker 8-OHdG [107].

Patchoulol also activates key DNA damage response pathways, including the phosphorylation of CHK1, CHK2, and H2A.X, while upregulating p21 and p53 levels. It inhibits NSCLC cell proliferation via the ATM/CHK2 and ATR/CHK1 pathways, promoting apoptosis with increased levels of Bax, cleaved caspase-3 and caspase-9, and decreased BCL-2 expression [107].

Furthermore, patchoulol addresses drug resistance in NSCLC by inhibiting the P-glycoprotein (P-gp) efflux pump, which typically reduces the efficacy of chemotherapy. It also impacts cancer stem cell markers, such as CD133 and CD44, which are associated with treatment resistance [150]. By targeting these mechanisms, patchoulol enhances chemotherapy effectiveness, particularly in drug-resistant NSCLC cells, highlighting its potential to improve treatment outcomes [151].

#### 4.2.2. Apoptosis Induction

Research using cell cultures and animal models has suggested that the cytotoxic and anticancer effects of pachypodol may involve several mechanisms. These include the induction of apoptosis and necrosis, activation of both specific and general immune responses, inhibition of cell cycle progression, and the release of β-endorphins into the bloodstream [81]. Based on these findings, research has explored the role of natural compounds in enhancing apoptosis as a part of cancer treatment strategies. For example, mistletoe extract has been used as complementary oncotherapy, particularly for its potential to induce apoptosis in cancer cells. Friedel et al. (2009) conducted a cohort analysis to assess the efficacy of mistletoe extract (Iscador) in patients with non-metastatic colorectal cancer [152]. The study found that the treatment decreased survival rates and induced apoptosis in several cell lines, including human leukemia K562, human plasmacytoma RPMI-8226, and murine lymphocytic leukemia L1210 cells [152]. This apoptotic process is facilitated by the activation of the intrinsic apoptosis pathway, as evidenced by the activation of caspase-9, JNK-1/2, and p38-MAPK, along with the inhibition of ERK-1/2 and PKB phosphorylation and the downregulation of Mcl-1 [81]. Moreover, anti-mutagenic protection is crucial for cancer prevention, with pachypodol showing considerable effectiveness against mutagenic agents [153].

#### 4.2.3. Effects on Tumors/Cancer Cells and the Immune System

##### Immunoregulatory Effect

The immune system plays a vital role in protecting an organism from pathogens and cancer. Patchoulol, a key compound in patchouli, has been shown to enhance immune functions by activating the mononuclear phagocytic system and suppressing excessive cellular immune responses [154]. PEO also exhibits immunomodulatory effects, notably through the stimulation of secretory immunoglobulin A (SIgA) production, which is crucial in mucosal immunity, defending against toxins, viruses, bacteria, and food antigens [155].

Further research on patchoulol’s immunomodulatory effects in Kunming mice found that it enhances the humoral immune response while suppressing cellular immunity [156]. Additionally, Xinxiang granule (XXG), which includes patchouli along with other herbs, has shown therapeutic benefits for allergic rhinitis and skin irritability [132,157]. PEO’s anti-allergic and anti-nociceptive properties have also been validated in mouse models [158].

Patchouli plants also contain non-volatile compounds, such as ombuin, licochalcone A, and 5,7-dihydroxy-3′,4′-dimethoxy flavanone, which exhibit cytotoxic effects. Licochalcone A has been shown to inhibit PI-PLC gamma 1, inducing terminal differentiation and monocyte production in promyelocytic leukemia cells (HL-60) [42]. Patchoulol inhibits HeLa cell proliferation, reduces cell differentiation, and induces apoptosis in colorectal cancer cell lines by inhibiting histone deacetylase 2, downregulating c-Myc, and activating the NF-κB signaling pathway [159,160]. Furthermore, α-bulnesene, a sesquiterpenoid in patchouli, demonstrates antiplatelet effects by inhibiting cyclooxygenase activity, thus reducing thromboxane production and preventing platelet aggregation [161,162].

### 4.3. Gastrointestinal Protective Activities

#### 4.3.1. Gastrointestinal Protective Activity

The aqueous extract of the patchouli plant has been shown to protect and preserve the membrane fluidity of intestinal epithelial cells by regulating the serum levels of tumor necrosis factor and nitric oxide (NO). This study establishes an experimental basis for utilizing patchouli for gastrointestinal protection following trauma or surgical procedures [163]. Li et al. investigated the metabolic profile of pogostone both in vitro and in vivo using LC–MS, revealing the significant gastroprotective effects of PEO against gastric ulceration [164]. The mechanisms underlying these anti-ulcerogenic effects are likely related to the stimulation of COX-mediated prostaglandin E2 (PGE2) production along with enhanced anti-inflammatory and anti-oxidant activities [90]. Additionally, PEO administration has been demonstrated to promote the repair of the intestinal epithelial ultrastructure, reduce intestinal permeability, and safeguard the intestinal mucosal mechanical barrier in a rat model [91].

#### 4.3.2. Antiemetic Activity

Patchouli has long been valued for its antiemetic effects and is traditionally used to address conditions such as dyspepsia, vomiting, diarrhea, and poor appetite. Research supports these uses, with studies showing that the n-hexane extract of patchouli leaves significantly reduces vomiting in young chicks induced by copper sulfate (CuSO_4_), outperforming other extracts, such as water, methanol, and chloroform, by 58.6%. This effect is primarily attributed to patchoulol, which is present in significant concentrations in patchouli and contributes to its antiemetic properties [165]. Additionally, patchoulol has demonstrated calcium ion (Ca^2+^) antagonist activity in vitro, suggesting that its ability to inhibit Ca^2+^ influx might alleviate excessive smooth muscle contractions in the digestive system, thereby reducing symptoms, such as diarrhea, nausea, and vomiting [166]. Other compounds from the n-hexane extract, including pogostol, stigmast-4-en-3-one, retusin, and pachypodol, also exhibit antiemetic effects [35].

Additionally, pogostone exhibits notable antiemetic properties and has proven effective in managing conditions such as diarrhea, anorexia, and dyspepsia, as well as in preventing vomiting and stimulating appetite. This therapeutic efficacy is partly linked to its high concentrations of elements, such as aluminum, vanadium, and manganese, which are believed to contribute to its beneficial effects [167].

#### 4.3.3. Defecation and Constipation

##### Anti-Peptic Ulcer Effect

Studies have shown that PEO influences bowel movements and constipation in two mouse models: one with flaccid bowel movements and another induced by a low-fiber diet [168]. Exposure to the aroma of PEO increased both fecal quantity and dry weight, likely due to the activation of olfactory neurotransmission pathways, which help relieve constipation [169].

Gastrointestinal ulcers, particularly in the stomach and duodenum, are complex disorders with widespread impact, often exacerbated by the chronic use of nonsteroidal anti-inflammatory drugs (NSAIDs) [166,170]. Pro-inflammatory cytokines and apoptotic factors are key in ulcer development [171]. Pogostone, a major component of PEO, has been shown to reduce oxidative stress and protect the gastrointestinal mucosa from ulcers induced by indomethacin. It upregulates anti-oxidant enzymes and reduces malondialdehyde (MDA) levels in rats while increasing prostaglandin E2 levels and modulating apoptosis-related proteins, which helps prevent cellular apoptosis [90,172].

Patchoulol, another component of PEO, similarly protects against gastric ulcers in rat models. Pretreatment with patchoulol reduces the severity of ethanol-induced gastric ulcers by enhancing anti-oxidant enzyme activity and decreasing MDA levels in gastric tissues [173]. Additionally, β-patchoulene, a compound found in patchouli, has gastroprotective effects, with its primary metabolite proving more effective than the parent compound in reducing ulcer size in rat models. This action is mediated through the modulation of the ERK1/2 and NF-κB signaling pathways [174,175].

Inflammatory bowel disease (IBD), which includes ulcerative colitis (UC) and Crohn’s disease, is a chronic gastrointestinal disorder marked by severe symptoms and mucosal ulceration [176]. Research indicates that PEO alleviates colonic damage and reduces disease activity markers, such as colonic myeloperoxidase (MPO), in rat models of UC induced by 2,4,6-trinitrobenzenesulfonic acid [177]. Patchoulol also reduces MPO levels and pro-inflammatory cytokines (IL-1β, IL-6, TNF-α) while increasing anti-inflammatory cytokines (IL-4, IL-10) in UC. It modulates mucin gene expression and tight junction proteins, which are vital for maintaining intestinal barrier integrity. Patchoulol also influences apoptosis-related proteins and reduces necrosis by downregulating receptor-interacting protein kinase 3 in acute colitis models [178]. Furthermore, patchoulol’s role as a pregnane X receptor (PXR) agonist and its involvement in PXR–NF-κB signaling pathways suggest new therapeutic options for treating colitis.

#### 4.3.4. Anti-Diarrheal Effect

Irritable bowel syndrome (IBS), especially the diarrhea-predominant subtype (IBS-D), is a prevalent functional gastrointestinal disorder [179]. Research shows that patchoulol inhibits spontaneous contractions of colonic longitudinal smooth muscle in a concentration-dependent manner, with an EC50 of 41.9 µM [180]. Additionally, patchoulol has proven effective in alleviating IBS-D symptoms in rat colon models [181]. These findings indicate that patchoulol plays a key role in the anti-diarrheal effects of patchouli, although the exact pharmacological mechanisms remain to be elucidated.

### 4.4. Blood-Related Activities

#### 4.4.1. Blood Coagulation and Fibrinolytic Activities

In vitro enzymatic assays have assessed various essential oils’ coagulation and fibrinolytic properties, including patchouli oil. These assays evaluate fibrin formation from fibrinogen through thrombin and the subsequent breakdown of fibrin by urokinase. The findings indicated that essential oils, such as chamomile, eucalyptus, and neroli, demonstrated coagulation and fibrinolytic activities. Conversely, PEO and citrus, pine, and frankincense oils exhibited pronounced hyper fibrinolytic activity, highlighting their potential efficacy in fibrin degradation [43].

#### 4.4.2. Antithrombotic Activities

##### Antihypertensive Activity

Hypertension is a major contributor to disability and early mortality, with over one billion people affected globally and approximately 9.4 million deaths annually [92]. Effective antihypertensive agents can significantly reduce the risk of cardiovascular events, such as stroke and coronary heart disease [182].

Patchoulol has been shown to exert antihypertensive effects through an endothelium-independent mechanism. It inhibits receptor-operated Ca^2+^ channels (ROCCs) and enhances α-receptor activity, both of which are essential for vasoconstriction [183]. Patchoulol modulates Ca^2+^ ion influx, reducing potassium chloride-induced contractions in the aorta [7,184]. By interfering with voltage-dependent Ca^2+^ channels (VDCCs) and membrane depolarization, patchoulol reduces the contraction response typically triggered by phenylephrine (PHE) and potassium chloride [185].

In addition, patchoulol decreases Ca^2+^-induced muscle contraction in endothelium-aortic rings and counteracts potassium ion depolarization. Combined with ruthenium red and heparin, patchoulol blocks Ca^2+^ ion channels, such as inositol triphosphate receptors (IP3R) and ryanodine receptors (RYR), which further reduces the effects of PHE [186]. This indicates that patchoulol inactivates ROCCs, limits Ca^2+^ influx, and enhances α1 receptor sensitivity, contributing to vasoconstriction [187,188].

Patchoulol also demonstrates vasodilatory effects by acting as a Ca^2+^ ion antagonist. It relaxes smooth muscle and dilates blood vessels by inhibiting Ca^2+^ influx across vascular smooth muscle cell membranes. This effect is mediated through the disruption of Ca^2+^ ion influx via the IP3R and RYR pathways [189]. Additionally, pocahemiketal B, a compound derived from PEO, exhibits significant vasorelaxant properties, effectively counteracting PHE-induced contractions in rat aortic rings with an EC50 value of 16.32 µM [188].

### 4.5. General Protective and Therapeutic Activities

#### 4.5.1. Anti-Oxidant Activity

Oxidative stress, characterized by elevated ROS levels, causes damage to lipids, proteins, and DNA due to free radical activity [93,94]. PEO, known for its free radical-scavenging properties, mitigates oxidative damage by preventing the oxidation of hexanal to hexanoic acid [14]. It protects brain cells from ROS-induced injury and cell death, suggesting potential benefits in neurodegenerative diseases [190]. Additionally, PEO combats photoaging by preserving skin integrity and preventing cellular ATP depletion and cytochrome C release caused by H_2_O_2_ [35]. It also neutralizes superoxide anions and hydroxyl free radicals and inhibits lipid peroxidation [191].

Pachypodol, a methoxy flavonoid, has demonstrated protective effects against oxidative damage in various tissues. In a study by Ijaz et al. (2022), pachypodol mitigated perfluorooctane sulfonate (PFOS)-induced testicular injury by scavenging ROS, improving spermatogenesis [124]. In the liver, pachypodol protects hepatocytes from oxidative stress by activating the Nrf2 via ERK1/2 signaling pathway [81].

Patchoulol, another compound from patchouli, protects intestinal epithelial cells (IEC-6) from heat shock-induced oxidative damage. Treatment with patchoulol increased HO1, and Nrf2 expression, enhancing cellular defense mechanisms and reducing markers of oxidative stress such as MDA [32]. However, high doses of patchoulol (above 80 ng) increase ROS production, suggesting a dose-dependent relationship with oxidative stress through the Nrf2-Keap1 pathway [192]. These findings underscore the potential of patchouli-derived compounds in reducing oxidative stress when administered in controlled amounts.

#### 4.5.2. Photoaging Activity

Skin aging, influenced by intrinsic and extrinsic factors, progressively disrupts the structural integrity and physiological function [193]. Photoaging is linked to increased activity of ROS-induced matrix metalloproteinases (MMPs) [194]. These MMP-mediated changes in the extracellular matrix commonly result in skin wrinkling, which is a key sign of premature aging. Pogostone administration has been demonstrated to alleviate both macroscopic and histopathological damage in UV-exposed mouse skin. This protective effect is achieved by boosting the activity of anti-oxidant enzymes, such as catalase (CAT), glutathione peroxidase (GSH-PX), and superoxide dismutase (SOD), while reducing MDA levels [195].

Furthermore, PEO demonstrated significant therapeutic effects on photoaged rat skin by modulating the p38-MAPK/ERK signaling pathway and associated apoptotic pathways. It inhibits the aberrant expression of MMP-1 and MMP-3 and prevents abnormal increases in markers such as Bax, Caspase-9, c-Fos, c-Jun, ERK1/2, MEK, MDA, p38-MAPK, Raf, and Ras, while maintaining levels of Bcl2, CAT, GSH-PX, and SOD [80,196].

#### 4.5.3. Analgesic and Anti-Inflammatory Activities

##### Analgesic Activity

Pain, a common symptom of many diseases, significantly disrupts normal physiological processes. It is triggered by the activation of nociceptors through various stimuli. Research has shown that patchouli can effectively reduce pain in mice subjected to acetic acid-induced writhing, indicating their analgesic potential in vivo [197]. Cyclooxygenase-2 (COX-2), an enzyme associated with inflammation, is often upregulated in response to inflammatory stimuli and certain cancers [198]. Notably, patchoulol increases the expression of COX-2 mRNA and protein both in vivo and in vitro [197].

Furthermore, the analgesic effects of patchoulol are linked to the mu-opioid receptor (MOR), a key player in pain modulation and relief through the opioid pathways [199]. Upregulation of MOR by patchoulol also affects intracellular Ca^2+^ levels, indicating its interaction with MOR [77,200,201]. These findings suggest that patchoulol’s dual modulation of COX-2 and MOR is a promising avenue for developing novel analgesic agents.

##### Anti-Inflammatory Activity

Inflammation, characterized by redness, swelling, heat, and pain, is a key defense mechanism against pathogens, driven by pro-inflammatory mediators, such as IL-1β, IL-6, NO, PGE2, and TNF-α [202]. Lipopolysaccharide (LPS), a component of Gram-negative bacteria, triggers inflammation by activating macrophages to produce these mediators [203].

β-patchoulene exhibits strong anti-inflammatory effects, particularly in LPS-stimulated RAW 264.7 macrophages, where it regulates the balance of pro- and anti-inflammatory cytokines. It reduces IL-1β, IL-6, and TNF-α levels while increasing IL-10 expression. β-patchoulene also inhibits COX-2 and iNOS activity, leading to decreased NO and PGE2 production, and suppresses NF-κB activity by increasing IκBα levels, preventing NF-κB translocation to the nucleus [100,204].

In vivo, β-patchoulene demonstrates dose-dependent anti-inflammatory effects in acute inflammation models, such as carrageenan-induced paw edema, xylene-induced ear edema, and acetic acid-induced vascular permeability. Histopathological analysis shows reduced cell infiltration, MDA levels, and MPO activity in inflamed tissues. Additionally, β-patchoulene lowers pro-inflammatory cytokines (IL-1β, IL-6, TNF-α), NO, and PGE2 and reduces vascular permeability by inhibiting mediators, such as serotonin, prostaglandins, and histamines [205,206].

Patchoulol oxide, an oxidative derivative of β-patchoulene, exhibits superior anti-inflammatory effects by reducing IL-1β, IL-12, MCP-1, and TNF-α expression at both mRNA and protein levels. It is more effective than β-patchoulene in decreasing NO and PGE2 production by inhibiting COX-2 and iNOS pathways [173]. PEO also limits leukocyte recruitment and modulates the NF-κB pathway to balance pro- and anti-inflammatory cytokines [207].

Methanol extracts of patchouli, containing phenolic compounds such as ombuine and pachypodol, show significant anti-inflammatory effects in various mouse models. Pachypodol inhibits NO production, a key player in organ edema pathogenesis, and pogostone reduces pro-inflammatory signaling by blocking p38-MAPK and JNK phosphorylation [80,81].

#### 4.5.4. Anti-Mutagenic Activity

The anti-mutagenic properties of pachypodol have been demonstrated in various assays. The umu test, using *S. typhimurium* TA1535/pSK1002, assessed its effectiveness against mutagenic chemicals, such as 2-(2-furyl)-3-(5-nitro-2-furyl) acrylamide (furylfuramide), and 3-amino-1,4-dimethyl-5H-pyrido [4,3-b] indole (Trp-P-1). The observed suppression effects in the umu test are likely attributed to the presence of a single methoxy group (–OCH_3_) at the 4′-position of the aromatic ring [35].

Additionally, pachypodol exhibited significant inhibition of SOS induction, showing more substantial effects at lower doses than furylfuramide. Specifically, at a 0.06 mol/mL concentration, pachypodol reduced over 80% of Trp-SOS-inducing P-1 activity. Additionally, pachypodol demonstrated more potent inhibition of the Trp-P-1-induced SOS response than furylfuramide. The Ames test with *S. typhimurium* TA100 further confirmed the anti-mutagenic properties of pachypodol against furylfuramide, Trp-P-1, and activated Trp-P-1 [87,208].

#### 4.5.5. Dermato-Protective Activity

PEO, at a concentration of 12%, has proven effective in managing skin infections and odors in patients with ulcers, abrasions, pressure sores, and torn skin. Its application significantly reduced healing time [46]. Furthermore, PEO helps preserve the structural integrity of UV-irradiated skin, prevents photoaging, and enhances the recovery of skin lesions [209,210].

#### 4.5.6. Insecticidal Activity

Controlling vectors and pests remains a significant challenge, especially in developing countries [211]. Pogostone, a major compound, exhibits acute toxicity to cockroaches (LC50 = 8.51 µg/adult) and inhibits dust mites (*Dermatophagoides farinae*) when extracted with petroleum ether [212]. Additionally, mosquito coils containing a 75:25 patchouli-to-valamus ratio significantly knocked down *Aedes aegypti* at a 7.5% concentration [213]. Pogostone also demonstrates larvicidal, antifeedant, pupicidal, and growth-inhibitory effects on pests such as *Spodoptera* spp., indicating its potential for crop protection [214].

Various studies confirm PEO’s larvicidal activity, with lethal concentration 50 (LC50) values ranging from 47.88 ppm against *A. albopictus* [215] to 254.79 ppm in different assessments [216]. Discrepancies in LC50 values highlight the need for standardized testing. Gokulakrishnan et al. (2013) found patchoulol to be a highly effective repellent, offering 100% protection against insects at 2 mg/cm^2^ for up to 280 min [217]. This underscores patchoulol’s potential as a superior natural repellent. In addition, PEO extract at 7% concentration provided over 90% protection for up to 6 h against mosquito bites [218], and undiluted oil provided complete repellency for 2 h [219]. These findings demonstrate the efficacy of essential oil-based repellents as alternatives to synthetic insecticides. However, further research is needed to identify the specific constituents of PEO responsible for its insecticidal activity and to understand its bio-insecticidal mechanisms better.

#### 4.5.7. Aromatherapeutic Activity

PEO is widely used in aromatherapy to relieve tension, insomnia, and anxiety [220]. Its distinct wine-like aroma is believed to act as an aphrodisiac, improve concentration, and enhance mental clarity [23]. Spiritually, patchouli incense creates a serene atmosphere [43]. Studies on healthy adults have shown that the fragrance of PEO reduces sympathetic activity, as evidenced by lower serum catecholamine levels and blood pressure [220].

In care settings, applying PEO in an aqueous cream five times daily has been shown to decrease dementia-related behaviors and improve mental alertness [43,221]. Additionally, PEO’s anti-inflammatory and cooling properties help alleviate menopausal symptoms, such as hot flashes and sweating [12,222]. A blend of patchouli with rose, ylang-ylang, jasmine, sandalwood, and vetiver is thought to balance thyroid function and promote harmonious energy flow [223].

PEO also has sedative and uplifting effects on cerebral function [224]. Inhalation of the oil has been shown to reduce physiological stress markers, such as blood pressure, pulse rate, and brain wave activity, suggesting both mood-enhancing and therapeutic properties [46,221]. These effects are primarily attributed to its primary compound, patchoulol [225]. Aromatherapy, including the use of PEO, is increasingly integrated into nursing care to manage stress, reduce chronic pain during cancer treatment, maintain skin integrity, and treat various psychiatric disorders [226].

#### 4.5.8. Aphrodisiac Activities

Patchoulol significantly increased aphrodisiac activity by increasing the production of norepinephrine by 23% when 40 mg/kg patchoulol was administered; however, there was also an increase in dopamine by 15% [227]. Administration of patchoulol increased NO [228]. Upregulation of NO from the endothelium and specific tissues induces the relaxation of blood vessels and smooth muscle in the penile; the relaxation of trabecular smooth muscle also lowers blood flow resistance accompanied by a decrease in vascular resistance, which influences blood flow in the penile, and decreased blood outflow triggers penile erection and engorgement with blood [22].

#### 4.5.9. Antidepressant Activity

A study investigating the antidepressant properties of patchouli plant extracts revealed significant findings with a 70% ethanol extract. When administered at doses of 500 mg/kg and 750 mg/kg, the extract notably reduced the immobility time in rats during both the despair swimming and tail suspension tests, compared to the vehicle control. Moreover, Dunnett’s test indicated that the 750 mg/kg dose significantly decreased spontaneous locomotor activity relative to the vehicle-treated group [35].

### 4.6. Metabolic and Obesity-Related Activities

#### 4.6.1. Gut Microbiota Modulatory Activity

Human gut microbiota plays a crucial role in maintaining homeostasis, influencing immune responses, nutrient absorption, and defense against pathogens [96,229]. An imbalance in the gut microbiota (dysbiosis) can lead to diseases such as IBD, obesity, and cancer [230,231].

Recent studies highlight that PEO, including key compounds such as pogostone, patchoulol, and β-patchoulene, positively influences gut health. These compounds strengthen the gut epithelial barrier, modulate macrophage activity, and improve gut microbiota composition. In mouse models, PEO has been shown to enhance gut microbiota diversity, reduce pro-inflammatory cytokines, and promote the transition of macrophages from the M1 to the M2 phenotype, supporting immune regulation and reducing inflammation [232].

PEO also aids in the production of antimicrobial proteins by upregulating goblet and Paneth cells, promoting mucin synthesis, and enhancing the secretion of lysozymes and defensins, which inhibit harmful bacteria [233,234]. Moreover, patchouli compounds improve gut barrier function by enhancing tight junction protein activity, which is crucial for maintaining epithelial integrity and preventing cancer development [235,236].

Additionally, PEO and its components inhibit adhesion molecules, such as intracellular adhesion molecule 1 (ICAM-1) and vascular cell adhesion molecule 1 (VCAM-1), which are involved in tumor progression, suggesting their potential role in cancer suppression [237,238]. Patchouli compounds also modulate macrophage polarization by reducing pro-inflammatory M1 markers (e.g., CXCL10 and iNOS) and promoting M2 markers (e.g., arginase-1, mannose receptor), further supporting cancer suppression and reducing inflammation [134].

Beyond their direct effects on immune modulation, patchouli compounds enhance gut microbiota health by promoting beneficial bacteria, such as *Akkermansia muciniphila,* and increasing short-chain fatty acid (SCFA) production, which plays a role in immune regulation and anti-inflammatory responses [239]. The modulation of the gut microbiota, combined with epithelial nourishment, suggests that PEO and its active compounds exhibit significant potential in preventing and managing inflammatory diseases and cancer.

#### 4.6.2. Antidiabetic Effect

Obesity is strongly associated with the development of type 2 diabetes and is a major risk factor for several metabolic disorders [240]. Recent studies have highlighted the impact of patchoulol on obesity management. Administration of patchoulol results in a significant reduction in body weight in high-fat diet (HFD)-induced obese mice, primarily by inhibiting adipogenesis and fat accumulation in adipocytes through enhanced β-catenin expression and activation [241]. Furthermore, inhalation of PEO at concentrations ranging from 0.3% to 1% markedly improves metabolic parameters in obesity-induced rats [242]. Chronic intake of high-fat diets is linked to various health issues, including non-alcoholic fatty liver disease (NAFLD), which affects approximately 38% of the population and is the most prevalent chronic liver disorder globally [243,244]. Recent studies have demonstrated that patchoulol exerts protective effects against HFD-induced hepatic steatosis in rats by alleviating endoplasmic reticulum stress and modulating the metabolism of very low-density lipoproteins (VLDL). Patchoulol also influences the expression of apolipoprotein B100, VLDL receptors, and microsomal triglyceride transfer protein, thereby improving liver health [245].

### 4.7. Other Activities

Secretory otitis media, marked by middle ear inflammation, tympanic effusion, and hearing loss, often arises from bacterial infections causing auditory tube dysfunction [246]. In guinea pig models, pogostone alleviates secretory otitis media-related hearing loss by reducing mucosal thickening and neutrophil infiltration, primarily inhibiting ICAM-1 and TNF-α expression in the ear mucosa [80]. Similarly, patchoulol has demonstrated anti-inflammatory effects in lung diseases, such as acute respiratory distress syndrome and chronic obstructive pulmonary disease (COPD) [247]. In mouse models of LPS-induced acute lung injury, patchoulol effectively suppressed pro-inflammatory cytokines IL-1β, IL-6, and TNF-α and inhibited the phosphorylation of IκB-α and p65 NF-κB [102,248]. Pogostone also offers protection against lung injury in COPD by decreasing inflammatory proteins p-IκBα and p-NF-κBp65 while increasing HO-1 and Nrf-2 expression, indicating a central role of the NF-κB signaling pathway in lung tissue preservation [80]. Additionally, patchoulol has shown cardiovascular benefits in atherosclerosis models, reducing atherosclerotic plaques, macrophage infiltration, and inflammatory cytokines in ApoE knockout mice [249].

## 5. Mechanisms of Action

**Figure 4:**
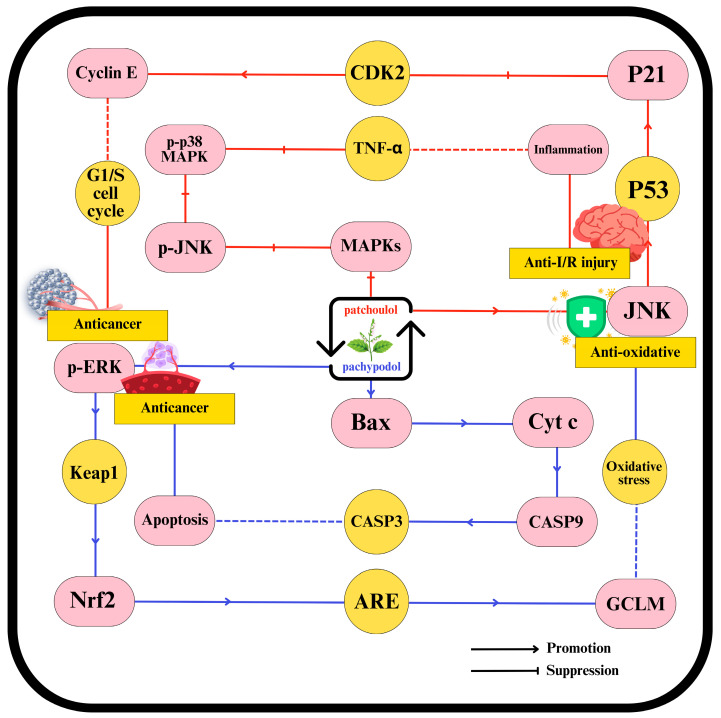
The molecular pathways are activated by patchoulol and pachypodol from patchouli leaves.

Recent experimental studies have expanded our knowledge of patchouli’s pharmacological properties, revealing a broad spectrum of therapeutic effects. These include anti-peptic ulcer [172], antimicrobial [62,122,250], anticancer [111], antioxidant [81,251], anti-inflammatory [7,248], ischemia-reperfusion injury protection [252], analgesic [253], antitumor [107,254], antidiabetic [255], antihypertensive [200], immunoregulatory [256], intestinal microecology [134], and anti-diarrheal activities [20]. Patchouli leaves-derived compounds, such as β-patchoulene, pachypodol, patchoulene epoxide, patchoulol, and pogostone, have shown protective effects in various organs, including the intestines, middle ear, and liver [36,257]. Advances in research methodology have allowed for an in-depth investigation of these monomeric components, with patchoulol and pachypodol being particularly well-studied. Their therapeutic efficacy is linked to the suppression of inflammation, oxidative stress reduction, apoptosis regulation, endoplasmic reticulum stress mitigation, and VLDL metabolism enhancement, as illustrated in Figure 4 [248].

Recent studies have identified multiple signaling pathways through which patchoulol exerts its therapeutic effects. For peptic ulcer treatment, patchoulol activates the PXR pathway while inhibiting the nuclear factor NF-κB pathway. These actions help maintain intestinal barrier integrity, reduce tryptophan catabolism, and inhibit cell death pathways. Key molecular changes include reduced levels of pro-inflammatory cytokines, downregulation of necroptosis-related proteins (MLKL and RIP3), decreased expression of indoleamine 2,3-dioxygenase-1 and tryptophan hydroxylase 1 (TPH-1), lowered anti-apoptotic Bcl-2, and pro-apoptotic Bax [248].

In diabetes treatment, patchoulol inhibits crucial pathways, such as activating transcription factor 6 (ATF6), inositol-requiring enzyme 1 (IRE1), protein kinase RNA-like ER kinase (PERK), and Wnt/β-catenin. This inhibition results in downregulation of ATF6, IRE1, and PERK; inhibition of very low-density lipoprotein receptor (VLDLR); increased apolipoprotein B 100 (apoB 100); enhanced microsomal triglyceride transfer protein (MTP) expression; increased Smad7 expression; and stabilization of β-catenin [258].

Patchoulol also mitigates ischemia-reperfusion injury by activating the ERK pathway and inhibiting the mitogen-activated protein kinase (MAPK) pathway [259]. Moreover, emerging research indicates patchoulol’s potential to prevent obesity and provide analgesia, though the underlying mechanisms are still being explored [258]. Pachypodol has been shown to protect hepatocytes from oxidative damage, likely through ERK1/2-dependent activation of Nrf2, which enhances endogenous antioxidant defenses [81]. Additionally, pachypodol exhibits cytotoxic and anticancer properties by inducing apoptosis and necrosis through mechanisms involving the activation of Bax, caspase-3, caspase-9, JNK-1/2, and p38 MAPK while inhibiting ERK-1/2 and PKB phosphorylation, and downregulating Mcl-1. It also offers significant anti-mutagenic protection, suggesting its potential for cancer prevention [81]. Despite these promising findings, further research is needed to elucidate other patchouli compounds’ molecular mechanisms and protein targets and address safety concerns through comprehensive preclinical studies. As existing research primarily relies on in vitro and small animal models, there is an urgent need for human clinical trials to validate these pharmacological effects and facilitate the development of patchouli-based traditional medicine formulations for broader applications.

## 6. Commercial Applications

### 6.1. Applications in Pharmaceuticals: Role in Medicine

Patchouli leaves have gained attention in the pharmaceutical industry due to their diverse pharmacological properties. The essential oil extracted from patchouli contains bioactive compounds, such as patchoulol, α-bulnesene, and pogostone, which exhibit anti-inflammatory, anti-mutagenic, and antioxidant effects [87,260]. In traditional Chinese medicine, patchouli is used in formulations, such as the Baoji and Houdan Pills, to treat inflammatory diseases [10,11]. Hopitan pills derived from patchouli address stomach discomfort, chest pain, diarrhea, and vomiting [167]. Globally, patchouli is used in various cultures for therapeutic purposes. In Uruguay, leaf infusions treat nervous disorders, while crushed leaves are applied topically to relieve rheumatism and arthritis [35]. Patchouli is also central to Ayurvedic formulations, such as Rasa, Guna, and Virya, and is recognized in several Asian countries for its antidepressant, antiseptic, and aphrodisiac properties [12,22,91]. These diverse applications suggest its potential for developing new therapeutic agents [42,261].

Recent research has focused on patchouli’s potential in treating skin infections and inflammatory conditions, as well as an adjunct therapy in cancer. PEO has demonstrated effectiveness against bacterial and fungal pathogens, such as *Cryptococcus neoformans*, *C. albicans*, herpes simplex, and methicillin-resistant *S. aureus* in AIDS patients, indicating its promise for developing new antibiotics and antifungal agents [103,262]. Additionally, PEO inhibits dermatophytes and fungi such as *Scopulariopsis brevicaulis* and *Chaetomium globosum*, suggesting its potential for treating pneumonia and chronic meningitis in 8–30% of AIDS patients [57].

### 6.2. Applications in Cosmetics: Role in Beauty and Skincare

PEO is employed extensively in the cosmetic industry due to its fragrant properties and ability to fixate aroma, particularly in the production of perfumes, aromatic incense, and other scented items [172,263]. It is a common ingredient in soaps, cosmetics, after-shave lotions, detergents, and various fancy products [2,264,265]. In addition to its aromatic appeal, PEO has numerous skin benefits [266]. Its anti-inflammatory and antiseptic properties effectively treat acne, eczema, and other skin conditions [36].

The regenerative properties of PEO contribute to wound healing and scar reduction, making it a valuable ingredient in anti-aging products. It enhances muscle, skin, and nerve contractions, which helps improve gum adherence to teeth, reduces hair loss, decreases muscle looseness, and prevents skin sagging [267]. Additionally, its antimicrobial properties promote skin health and hygiene, further supporting its use in cosmetic formulations [268]. The versatility of PEO was also demonstrated by its effectiveness in spray gels and microemulsions, offering both aesthetic and therapeutic benefits in beauty and skincare applications [269].

### 6.3. Other Industries: Additional Commercial Applications

In addition to pharmaceuticals and cosmetics, patchouli has applications in several industries. PEO is sometimes used as a flavoring agent in the food and beverage industry because of its distinctive taste and aroma [45]. PEOs are recognized by the Food and Drug Administration (FDA) as a safe, natural flavoring component for human consumption. They are commonly used as flavoring agents in various food products, including meat and meat products, gelatin, frozen dairy desserts, and non-alcoholic and alcoholic beverages [270,271]. The agricultural sector utilizes patchouli as a natural pesticide and insect repellent because of its potent insecticidal properties [272]. The bioactive compounds in patchouli exhibit selective toxicity, minimizing adverse effects on higher organisms and the environment [272,273]. Moreover, PEO is employed in aromatherapy for its calming and grounding effects, which help alleviate stress and anxiety. The fragrance industry also uses patchouli leaves to form incense and scented candles, capitalizing on their long-lasting aroma [274].

Additionally, manufacturers have integrated the aroma of patchouli into air fresheners, paper towels, laundry detergents, and other similar products to enhance their fragrance [275]. Overall, the multifaceted applications of patchouli leaves in pharmaceutical, cosmetic, and other industries highlight their significant commercial potential and contribution to various sectors. Advancements in extraction methods, chemical analyses, and economic studies have enhanced the commercial value of patchouli leaves, positioning them as versatile resources in the commercial sector [276].

## 7. Challenges and Limitations

### 7.1. Phytochemical and Genetic Variabilities

Despite significant advancements in the study of the patchouli plant, and specifically its leaves, several challenges and limitations have affected our understanding and utilization of this valuable plant resource. The phytochemical composition of patchouli leaves can vary significantly owing to geographical and environmental factors, including geographical location, climate, soil conditions, and cultivation practices, all of which influence the consistency and efficacy of therapeutic products derived from leaves [16]. Genetic variability among different strains or cultivars of patchouli contributes to diverse profiles of its active compounds, further leading to inconsistent therapeutic effects. Mechanistic studies are needed to elucidate the molecular mechanisms underlying the pharmacological effects of patchouli, which are not well understood [48]. Furthermore, rigorous, large-scale clinical trials are scarce to substantiate the therapeutic claims associated with patchouli extracts [277].

### 7.2. Methodology and Quality Control

Methodological challenges in current research on patchouli leaves include a lack of standardized extraction methods [278]. The techniques for extracting active compounds from patchouli leaves can influence the quality and concentration of phytochemicals, highlighting the need for standardized extraction protocols to ensure consistency. The distillation process for extracting PEO is time-consuming, primarily because of the high sesquiterpene content of the leaves. This extended distillation time can affect the efficiency of oil extraction processes and may require innovative approaches to streamline production while maintaining the oil quality [45].

Additionally, some studies suffer from inadequate experimental design, including insufficient control groups and small sample sizes, which can compromise the validity and reproducibility of the findings. Another methodological challenge is the reliance on conventional analytical techniques that may not detect all bioactive compounds, highlighting the need for more advanced methods [21]. Quality assurance is also crucial, as contamination with pesticides, heavy metals, or adulterants can pose significant health risks, emphasizing the importance of ensuring the quality and purity of patchouli extract. Addressing these methodological limitations is crucial for enhancing the scalability and sustainability of PEO production [45].

### 7.3. Regulatory, Standardization and Safety Issues

Regulatory, standardization, and safety challenges further complicate the commercialization of patchouli products. Obtaining regulatory approval for therapeutic use involves rigorous testing and compliance with safety standards, making the process time-consuming and expensive. A significant issue is the lack of standardized guidelines for the quality control of patchouli leaf products, leading to variable product quality and efficacy [279]. Regulatory challenges include inconsistencies in legislative frameworks governing the use and distribution of herbal products, which can affect their marketability and acceptance. These regulatory and standardization challenges hinder the consistent therapeutic use of patchouli products [280]. Finally, comprehensive toxicological studies are necessary to ensure the safety of patchouli-based products, as potential side effects, interactions with other drugs, and long-term safety profiles must be thoroughly investigated. Ensuring consistency in product quality and adherence to regulatory standards are essential for market acceptance and consumer trust. Harmonizing regulations and establishing industry standards for patchouli products can help overcome these challenges and promote market growth [281].

### 7.4. Bioavailability and Efficacy

Another challenge is the low bioavailability of certain phytochemicals in patchouli leaves, which means that they are not easily absorbed or metabolized by the human body, and enhancing their bioavailability is essential for the effective therapeutic use of these compounds [282]. Variations in the composition of bioactive compounds, such as patchoulol, across different plant parts and growth stages can affect the overall efficacy of patchouli-based products [283]. Additionally, the efficacy of these extracts can be influenced by factors such as extraction method, formulation, and individual patient differences [19]. Finally, determining the optimal dosages that are both effective and safe is complex and requires extensive clinical testing and validation. Achieving consistent bioavailability and therapeutic effects requires a deeper understanding of the factors influencing plant compound accumulation and activity. Developing strategies to enhance bioavailability and ensure consistent efficacy is crucial for maximizing patchouli leaves’ medicinal and commercial value [284].

Although patchouli leaves offer various commercial applications, challenges and limitations impede their full utilization. Addressing research gaps, overcoming methodological issues, navigating regulatory complexities, and optimizing bioavailability are essential steps in advancing the understanding and commercialization of patchouli products [285]. By addressing these challenges through interdisciplinary research and collaborative efforts, the potential of patchouli leaves as a valuable herbal resource can be fully realized.

## 8. Future Perspectives

### 8.1. Emerging Trends: New Directions in Research

Emerging trends in research on patchouli leaves indicate a growing interest in understanding their comprehensive phytochemical profiles and their health implications. Advances in analytical techniques, such as MS and HPLC, have facilitated more detailed studies of the chemical constituents of patchouli [46]. There is a growing emphasis on the genetic engineering of patchouli plants to optimize the production of specific bioactive compounds. The use of omics technologies, including genomics, proteomics, and metabolomics, has become increasingly prevalent and provides deeper insights into the metabolic pathways involved in the biosynthesis of these compounds [286]. By leveraging biotechnological approaches, researchers aim to enhance the sustainable production of PEO components, opening new avenues for industrial applications and therapeutic uses [287].

### 8.2. Potential for New Applications: Opportunities for Novel Therapeutic Uses

The potential for new applications of patchouli extends beyond their traditional use. Research currently explores its efficacy in treating various conditions, including inflammatory and neurodegenerative diseases. The antioxidant and anti-inflammatory properties of patchouli extracts suggest their potential use in managing conditions such as arthritis and Alzheimer’s disease [288]. Additionally, the antimicrobial properties of patchouli are being investigated to develop novel antibiotics and antifungal agents. Understanding the metabolic dynamics of patchouli leaves can lead to the development of targeted interventions to modulate bioactive compound production for specific applications in medicine, cosmetics, and other fields [39].

### 8.3. Directions for Future Studies: Suggestions for Further Research

Future studies on patchouli should address several key areas to advance this field. First, more comprehensive phytochemical analyses are needed to identify and quantify bioactive compounds in different parts of the plant [70]. Second, in-depth mechanistic studies are essential to elucidate the pathways through which these compounds exert their pharmacological effects [154]. Third, well-designed clinical trials are crucial to validate the therapeutic potential of patchouli extracts in humans [132]. Additionally, research should focus on optimizing extraction and formulation techniques to improve the consistency and efficacy of patchouli-based products [14]. Finally, interdisciplinary collaborations integrating pharmacology, molecular biology, and bioinformatics could accelerate the discovery of new applications and enhance our understanding of the therapeutic potential of patchouli [132]. In conclusion, patchouli research has immense potential for uncovering new trends, applications, and directions for further studies. By leveraging advances in metabolic engineering, metabolomics, genetic analysis, and pharmacological research, researchers can unlock patchouli leaves’ total therapeutic and commercial value, paving the way for innovative products and interventions in diverse industries [286,289].

## 9. Conclusions

This review comprehensively summarizes patchouli leaves’ phytochemical diversity and pharmacological potential, highlighting their rich profile of volatile and non-volatile bioactive compounds. Our evaluation showed that patchouli leaves contain various bioactive compounds, including flavonoids, sesquiterpenes, glycosides, phytosterols, alcohols, organic acids, and phenolic acids, all contributing to their therapeutic properties. These compounds exhibit significant anti-oxidant, anti-inflammatory, anticancer, antidepressant, diuretic, antithrombotic, cytotoxic, antimicrobial, and insect-repellent properties, indicating a broad spectrum of potential medical applications. These findings highlight the potential of patchouli leaves as a versatile medicinal resource with broad-spectrum applications. However, despite their traditional use and promising pharmacological properties, there is a critical need for the scientific validation and standardization of these bioactive compounds. Future studies should prioritize elucidating precise mechanisms of action, optimizing extraction methodologies, and conducting robust clinical trials to establish the efficacy and safety of patchouli-derived therapeutics. Many fundamental and applied aspects of the bioactive compounds in patchouli remain underexplored, highlighting the urgent need for further research on patchouli leaves to understand their potential fully. By bridging traditional medicine with modern pharmacology, this review highlights the opportunities for innovative healthcare solutions and the development of novel, effective, and sustainable therapeutic agents.

## Figures and Tables

**Table 1:** The structures of some of the volatile chemical constituents in patchouli leaves.

Compound Name	Formula	Part	Analytical Method	Reference
Monoterpenes				
(−)-camphor	C_10_H_16_O	aerial part	GC×GC–TOF-MS	[41]
β-phellandrene	C_10_H_16_	leaves	GC×GC–TOF-MS	[41]
β-pinene	C_10_H_16_	aerial part	GC×GC–TOF-MS	[41,49,50]
Sesquiterpenes				
α-bulnesene	C_15_H_21_	leaves	GC-MS	[41,49,51]
α-, β, δ-guaiene	C_15_H_24_	leaves	GC-MS, GC	[49,52]
cycloseychellene	C_15_H_24_	leaves	GC-MS	[49]
limonene	C_10_H_16_	aerial part	GC-MS, GC	[49]
globulol	C_15_H_26_O	aerial part	GC-TOF-MS, GC	[53]
α-, β -patchoulene	C_15_H_24_	leaves	GC-TOF-MS	[49,54]
patchouli alcohol	C_15_H_26_O	aerial part	GC-MS, GC	[49]
nortetrapatchoulol	C_14_H_24_O	aerial part	GC-TOF-MS, GC-MS	[49]
pogostol	C_15_H_26_O	aerial part	GC×GC-TOF-MS	[49]
α-humulene	C_15_H_24_	leaves	GC-MS	[49]
seychellene	C_15_H_24_	leaves	GC-MS	[49,55,56]
*trans*-caryophyllene	C_15_H_24_	leaves/shoot	GC-MS	[57]
α-elemenone	C_15_H_22_O	leaves	GC×GC–TOF-MS	[41]
(-)-α-selinene	C_15_H_24_	leaves	GC-MS	[50]
β-caryophyllene	C_15_H_24_	leaves	GC-MS	[41,49,50]
δ-patchoulene	C_15_H_24_	leaves	GC-MS, GC, NMR	[49,52]
γ-gurjunene	C_15_H_24_	leaves	GC-MS	[50,58]
δ-elemene	C_15_H_24_	leaves	GC-MS	[49,59]
*cis*-β-elemene	C_15_H_24_	leaves	GC-MS	[49]
β-selinene	C_15_H_24_	leaves	GC-MS	[49,50]
β-elemene	C_15_H_24_	leaves	GC-MS, NMR	[59]
germacrene D	C_15_H_24_	leaves	GC-MS, GC	[49,58]
zizanal	C_15_H_22_O	leaves	GC-MS	[49]
Alcohols				
norpatchuolenol	C_14_H_22_O	leaves	GC-MS	[49]
Ketones				
pogostone	C_12_H_16_O_4_	leaves	GC-MS	[41,49]
3-iso-thujopsanone	C_15_H_24_O	leaves	GC-MS	[49]

Note: In the referenced studies, aerial parts refer to all above-ground parts of the plant, including leaves, stems/shoots, and flowers/inflorescences (if present). For consistency with the focus of this review, data related to aerial parts are interpreted in the context of patchouli leaves, the primary medicinal component.

**Table 2:** The structures of some of the non-volatile chemical constituents in patchouli leaves.

Compounds Name	Formula	Part	Analytical Method	Reference
Flavonoids				
apigenin	C_15_H_10_O_5_	aerial part	HPLC-DAD	[41,73]
pachypodol	C_16_H_12_O_6_	aerial part	CC	[41,73]
3,5-dihydroxy-7,4′-dimethoxy-flavone	C_17_H_14_O_6_	aerial part	HPLC-DAD	[74]
rhamnetin	C_16_H_12_O_7_	aerial part	HPLC-DAD	[73]
4′,5-dihydroxy-3,3′,7-trimethoxyflavone	C_18_H_18_O_7_	aerial part	HPLC-DAD	[74]
5-hydroxy-3,3′,4′,7-tetramethoxyflavone	C_18_H_18_O_6_	aerial part	HPLC-DAD	[74]
5-hydroxy-3,4′,7-trimethoxyflavone	C_17_H_16_O_6_	aerial part	HPLC-DAD	[74]
5-hydroxy-7,3′,4′-trimethoxyflavanone	C_17_H_16_O_6_	aerial part	HPLC-DAD	[74]
4′,5,7-trihydroxyflavone	C_15_H_10_O_5_	aerial part	HPLC-DAD	[74]
Glycosides				
isoacteosides	C_29_H_36_0_15_	aerial part	TLC, HPLC, HPLC-Q-TOF-MS	[62,75]
7R-campeoside II	C_29_H_36_O_16_	aerial part	HPLC	[76]
7S-campeoside II	C_29_H_36_O_16_	aerial part	HPLC	[76]
verbascoside	C_29_H_36_O_15_	aerial part	HPLC	[77]
osmanthuside B	C_29_H_36_O_13_	aerial part	HPLC-DAD	[76]
actinoside	C_36_H_44_O_20_	leaves	HPLC-DAD	[14]
pedicularioside G	C_17_H_26_O_11_	aerial part	HPLC-DAD	[77]
crenatosides	C_29_H_34_O_15_	aerial part	TLC, HPLC	[75]
2″,3″-O-acetylmartynoside	C_35_H_46_O_17_	aerial part	HPLC-DAD	[76]
3′-methoxyisocrenatoside	C_30_H_34_O_16_	aerial part	HPLC-DAD	[76]
isocrenatoside	C_29_H_34_O_15_	leaves	CC	[41,52]
acteoside	C_29_H_36_O_15_	aerial part	TLC, HPLC, HPLC-Q-TOF-MS	[75,78]
Triterpenoids				
oleanolic acid	C_30_H_48_O_3_	aerial part	NMR, IR, MS, UV	[41]
friedelin	C_30_H_50_O	aerial part	NMR, IR, MS, UV	[41]
epifriedelinol	C_30_H_52_O	aerial part	NMR, IR, MS, UV	[41]
methyl oleanolate	C_31_H_50_O_3_	aerial part	NMR, IR, MS, UV	[41]
Other compounds				
lariketoester	C_33_H_39_O_10_	aerial part	HPLC	[76]
isolariketoester	C_33_H_39_O_10_	aerial part	HPLC	[76]
cytosporone W	C_12_H_15_O_5_	aerial part	HPLC	[76]
cytosporone V	C_13_H_16_O_5_	aerial part	HPLC	[41]

Note: In the referenced studies, aerial parts refer to all above-ground parts of the plant, including leaves, stems/shoots, and flowers/inflorescences (if present). For consistency with the focus of this review, data related to aerial parts are interpreted in the context of patchouli leaves, the primary medicinal component.

**Table 3:** The pharmacological activities of the volatile chemical constituents in patchouli leaves.

Components	Chemical Group	Pharmacological Activity
α-pinene	Monoterpenes	Antifungal [97] Anticancer [98] Respiratory diseases [99]
β-patchoulene	Sesquiterpene	Anti-inflammatory, Antitumor [100]
guainine-sesquiterpenoids		Anti-hypoglycemic [101]
patchouli alcohol		Anticancer [102] Antibacterial [13,103,104] Anti-oxidative [105,106] Antitumor [107]
pogostol		Anti-influenza [108]
pogostone	Ketone	Anti-inflammatory [109,110] Anticancer [111,112] Anti-apoptosis [113] Antimicrobial [62] Antifungal [114,115]

**Table 4:** The pharmacological activities of the non-volatile chemical constituents in patchouli leaves.

**Components**	**Chemical Group**	**Pharmacological Activity**
apigenin	Flavonoid	Anticancer [116,117] Apoptosis [118] Aphrodisiac [22]
rhamnetin		Apoptosis [118] Anti-oxidant [119] Antitumor [120]
5,4′-dihydroxy-7-methoxyflavone		Antiproliferative [121] Apoptosis [121]
pachypodol		Antimicrobial [122] Apoptosis [123] Anti-oxidative [124] Anti-inflammatory [7] Anti-apoptotic [7] Anticancer [123] Antiemetic [14]
5,7-dihydroxy-3,4-dimethoxyflavanone		Anti-apoptotic [125] Antiviral activity [125]
ombuine		Anticancer [14]
licochalcone		Anticancer [14,126]
isoacteoside	Glycoside	Anti-inflammation [126] Antiviral activities [125]
acteoside		Anti-inflammation [126]
verbascoside		Anti-apoptotic [125] Antiviral activity [125]

## Abbreviations

The following abbreviations are used in this manuscript:

BCL-2	B-cell lymphoma 2
cAMP	cyclic adenosine monophosphate pathway
CAT	catalase
CDK 1	cyclin-dependent kinase 1
CDK4	cyclin-dependent kinase 4
COPD	chronic obstructive pulmonary disease
COX-1	cyclooxygenase 1
DNA	deoxyribonucleic acid
DSS	dextran sodium sulfate
FDA	food and drug administration
GC/MS	gas chromatography mass spectrum
GCLC	glutamate cysteine ligase comprising of catalytic subunit
GCLM	glutamate-cysteine ligase, modifier subunit
GES-1	gastric epithelial cells
GSH	glutathione
GUs	gastric ulcers
HCT116	human carcinoma cells and large intestine cells
HDAC	histone deacetylase
HFD	high-fat diet
HIV/AIDS	human immunodeficiency virus/acquired immunodeficiency syndrome
HO-1	heme oxygenase-1
HPLC-DAD	high-performance liquid chromatography-diode array detection
IBD	inflammatory bowel disease
ICAM-1	intercellular adhesion molecule 1
IFN-γ	interferon gamma
IkB-α	inhibitor of kappa B alpha
IL-1β	interleukin-1 beta
IP3R	inositol triphosphate receptors
KEAP1	kelch-like ECH-associated protein 1
LC50	lethal concentration 50
M1	macrophages markers-1
MCP-1	monocyte chemotactic protein-1
MDA	malondialdehyde
MIC	minimum inhibitory concentration
MMPs	matrix metalloproteinases
MOR	mu-opioid receptor
MR	mannose receptor
NAD(P)H	nicotinamide adenine dinucleotide (phosphate)
NAFLD	non-alcoholic fatty liver disease
NF-κB	nuclear factor-kappa B
NMR	nuclear magnetic resonance
NO	nitrous oxide
NQO1	quinone oxidoreductase 1
Nrf2	nuclear factor erythroid 2-related factor 2
NSAIDs	nonsteroidal anti-inflammatory drugs
NSCLC	non-small cell lung cancer
PCLE	patchouli ethyl acetate
PCLH	patchouli hexane
PFOS	perfluoro octane sulfonate
PGE2	prostaglandin E2
P-gp	P-glycoprotein
PHE	phenylephrine
PXR	pregnane X receptor
ROCCS	rapid overlay of chemical conformations and structures
ROCCS	receptor-operated Ca^2+^ channels
ROS	reactive oxygen species
RYR	ryanodine receptors
SCFA-GPR	short-chain fatty acid-G protein-coupled receptor
SCLC	small cell lung cancer
SOD	superoxide dismutase
SOM	secretory otitis media
TLC	thin-layer chromatography
TNF-α	tumor necrosis factor-α
TOF	time-of-flight
UC	ulcerative colitis
VCAM-1	vascular cell adhesion molecule 1
VCR	vincristine
VDCCS	voltage-dependent calcium channel subunit
VLDL	very low-density lipoproteins
β-PAE	β-patchoulene
